# Keap1/Nrf2 signaling pathway participating in the progression of epilepsy via regulation of oxidative stress and ferroptosis in neurons

**DOI:** 10.1016/j.clinsp.2024.100372

**Published:** 2024-05-10

**Authors:** Dandan Wang, Yunmei Cui, Fan Gao, Weiwei Zheng, Jinzi Li, Zhemin Xian

**Affiliations:** a Department of Ultrasound Medicine, Affiliated Hospital of Yanbian University, Yanji, Jilin, China; b Department of Pediatrics, Affiliated Hospital of Yanbian University, Yanji, Jilin, China

**Keywords:** Keap1/Nrf2 signaling pathway, Oxidative stress, Ferroptosis, Epilepsy, Neuronal cell

## Abstract

**Objective:**

This study aims to analyze the relationship between the Kelch-like ECH-associated protein 1 (Keap1)/Nuclear factor-erythroid 2-related factor 2 (Nrf2) and Epilepsy (EP), as well as its mechanism of action.

**Methods:**

Thirty Wistar rats were divided into a control group (without treatment), a model group (EP modeling), and an inhibition group (EP modeling + intervention by Keap1/Nrf2 signaling pathway inhibitor ATRA) and subject to Morris water maze experiment. Then, the expression of Oxidative Stress (OS) markers, ferroptosis-associated proteins and Keap1/Nrf2 pathway in rat hippocampus was measured. In addition, rat hippocampal neuronal cell HT22 was purchased and treated accordingly based on the results of grouping, and cell proliferation and apoptosis in the three groups were determined.

**Results:**

Compared with rats in the model group, those in the inhibition group showed shorter escape latency and an increased number of platform crossings (p < 0.05). Significant OS and neuron ferroptosis, increased apoptosis rate, elevated Keap1 expression, and decreased Nrf2 expression were observed in the model group compared to the control group (p < 0.05). The inhibition group exhibited notably improved OS and ferroptosis, as well as enhanced neuronal viability (p < 0.05).

**Conclusion:**

Inhibition of the Keap1/Nrf2 pathway can reverse the OS and neuron viability in EP rats.

## Highlights

• Role of the Keap1/Nrf2 pathway in epilepsy.
• Effects of the Keap1/Nrf2 pathway on ferroptosis and oxidative stress in neuronal cells.
• Effect of Keap1/Nrf2 pathway on neuronal cell activity in epileptic rats.

## Introduction

Epilepsy (EP) is a chronic brain disorder characterized by recurrent seizures, believed to be primarily caused by abnormal electrical discharges in the brain.^1^ Epidemiological studies by the World Health Organization (WHO) have shown that EP affects the health of over 70 million people worldwide, with approximately 6‒9 million patients in China alone, and an average of 400,000‒600,000 new cases each year.^2^^,^^3^ The repeated seizures of EP not only seriously affect the normal lives of patients, but also disrupt their neurological function, leading to cognitive disorders, cerebral atrophy, brain necrosis, and loss of the ability to perform daily activities in severe cases.^4^ Due to the complex and diverse pathogenesis of EP, the existing treatment options in clinical practice are generally inefficient and cannot cure the disease.^5^ Accelerating and deepening the research on anti-EP drugs is of great significance to safeguard the health of patients with EP.

In recent years, the role of the Kelch-like ECH-associated protein 1 (Keap1)/Nuclear factor-erythroid 2-related factor 2 (Nrf2) signaling pathway in nervous system disorders has received extensive clinical attention.^6^^,^^7^ As a currently recognized Oxidative Stress (OS) regulatory pathway, the Keap1/Nrf2 pathway has been proven to affect the progression of Parkinsonism by mediating OS and mitigating neuron ferroptosis.^8^^,^^9^ OS damage and neuron ferroptosis have been repeatedly confirmed as typical pathological features in studies regarding the pathogenesis of EP.^10^^,^^11^ These findings strongly suggest that the Keap1/Nrf2 pathway may play an important role in the progression of EP. A recent study by Kishore M et al. suggested that the development of EP might be inhibited by modulating the Keap1/Nrf2 axis,^12^ but they did not experimentally validate this. In addition, a study by Hu QP et al. found that Genistein protects EP-induced brain injury by regulating the JAK2/STAT3 and Keap1/Nrf2 signaling pathways in the developing rats,^13^ which further confirmed the important role of Keap1/Nrf2 in EP. However, although these studies have preliminarily confirmed the relationship between Keap1/Nrf2 and EP, the mechanism of Keap1/Nrf2′s effect on EP needs to be studied and analyzed in greater depth.

Therefore, the present study analyzed the effect of the Keap1/Nrf2 pathway on OS and neuronal cell iron death in EP by constructing a rat model of EP. This study is the first of its kind to observe the effects of the Keap1/Nrf2 pathway on EP by regulating its expression. These results not only confirm the mechanism of Keap1/Nrf2 effects on EP but also provide a reference for the future clinical development of new anti-EP drugs.

## Materials and methods

### Animal data

Thirty 6-week-old male Wistar rats of Specific Pathogen Free (SPF) grade, weighing 180‒200g, were purchased from Nanjing Immunophage Biotech Co., Ltd., with the certificate number SYXK (Su) 2022‒0052. The study has been approved by the Animal Ethics Committee of the hospital (2020192). The experimental process was performed in strict accordance with the principle of the 3Rs (Reduction, Replacement and Refinement) and the ARRIVE Guide.

### Animal grouping and modeling

After one week of adaptive feeding, the rats were randomized into three groups, including a control group and two EP modeling groups. Rats in the latter two groups received intraperitoneal injections of 40 mg/kg pentylenetetrazol (Sigma, USA), twice a day, for one month. At 1h after the last dose, the behavior of the rats was observed and evaluated using the Racine scale.^14^ The modeling was considered successful when the rats exhibited Grade V seizures (generalized tonic-clonic activity, with loss of limb control) three consecutive times. The injection frequency of pentylenetetrazol was then changed to every three days to maintain the EP model. Of the two EP modeling groups, one was randomly selected as the inhibition group, where rats received intraperitoneal injection of 10 mg/kg ATRA (MedChemExpress, USA), an inhibitor of the Keap1/Nrf2 signaling pathway, once a day, for four weeks. The other group was considered as the model group, in which rats received an equal amount of normal saline.

### Behavior test

After grouping, the Morris water maze experiment was carried out.^15^ A circular water pool with dimensions of 40 × 90 × 30 was established, and the temperature was maintained at 22°‒26°C The pool was divided into four quadrants, and a circular glass platform was placed in the middle of the third quadrant below the water surface by 2 cm. Four entry points were marked on the pool walls. Rats were placed in the pool, and the time they spent finding the platform within the 90s (escape latency) was recorded. Rats failed to find the platform and were placed on it for 10s. All rats were trained for 5 days, twice a day. On the 6^th^ day, the formal experiment began, and the escape latency and the number of platform crossings were recorded.

### Enzyme-linked immunosorbent assay (ELISA)

After behavior tests, all the rats were executed in an anesthetized state by cervical dislocation. The hippocampus was obtained and homogenized in tissue lysate. The supernatant was then obtained by centrifugation. The levels of Glutathione (GSH), Superoxide Dismutase (SOD), and Malondialdehyde (MDA), as well as iron (Fe) content in the hippocampus, were measured using an ELISA kit (Beijing TransGen Biotech Technology, China).

### Polymerase chain reaction (PCR)

Total RNA was extracted from the hippocampus using a TRIzol extraction kit (Thermo Fisher Scientific, USA), verified by a UV spectrophotometer (Thermo Fisher Scientific, USA) for purity, and reversely transcribed into cDNA. Then, a PCR reaction was performed based on the primer sequences of Keap1, Nrf2, and GAPDH (designed by Shanghai Dynegene Technologies Co., Ltd., as contracted). The reaction conditions were as follows: 90°C for 120s (1 cycle), 90°C for 50s, 50‒65°C for 50s, and 70°C for 60s (50 cycles). The relative expression of Keap1 and Nrf2 was calculated using the 2^−△△CT^ method, with the primer sequences listed in Table 1.

**Table 1:** Sequence of primers.

	Keap1	Nrf2	β-actin
**F (5’-3’)**	CATGAAGCATCGGCGAAGTG	GGAGACTGAGGAAAAGACA	GAACGGAGAAGGTGACAGCA
**R (5’-3’)**	GAACAAAAACCGGCCTGACC	TTTCCGATGGGATGTGTGGG	GGCTTTTGGGAAGGCAAAGG
**Primer length (bp)**	562	260	163

## Western blot

The total protein of rat hippocampus was extracted with tissue lysis buffer (abcam, USA), and the protein concentration was determined by Bicinchoninic Acid (BCA) assay (abcam, USA). Fifty μg of protein was transferred to PVDF membrane (Sigma-Aldrich, USA) by electrophoresis, sealed with 10% skimmed milk powder at room temperature for 1h, then added with Keap1, Nrf2, GPX4, PTGS2, SLC7A11, LC3-II, Beclin1 and GAPDH primary antibody (1:500) (abcam, USA) for incubation overnight at 4°C. The membrane was washed the next day and added with goat anti-rabbit IgG secondary antibody (1:1,000) (abcam, USA) for incubation at room temperature for 1h. Then, quantitative analysis was performed by Image J software after color development by Enhanced Chemiluminescence (ECL) (Sigma-Aldrich, USA).

### Cell data

Mouse hippocampal neuronal cell HT22 (BNCC358041) was purchased from BeNa Culture Collection, Beijing, and cultured in a supporting medium (90% DMEM-H + 10% FBS). Cells that had been passaged 5 times were used for subsequent experiments.

### Cell grouping and intervention

HT22 cells were randomized into a control group (normally cultured without treatment), and a model group (cultured with medium containing glutamate to induce EP model, the cultured cells were seeded onto a 6-well plate at a density of 3.4 × 104 cells/well. After overnight incubation, cells were treated with a sample to be tested for an hour followed by 2.5 mM glutamate insult)^16^ and an inhibition group (cultured with medium containing 23 μmoL/L ATRA after EP model establishment).^17^

### Cell Counting Kit-8 (CCK-8)

When the cell confluence in each group reached 80%, the cells were washed with PBS 3 times, digested and resuspended, and inoculated into a 96-well plate at 3‒6 × 10^3^ cells/well, with 4 replicate wells in each group. At 24h, 48h, and 72h of culture, respectively, one well was selected for the addition of 10 μL of CCK-8 (MedChemExpress, USA) solution and measured for the Optical Density (OD) at 450 nm using a microplate reader after 2h of culture, and corresponding cell growth curves were plotted.

### Flow cytometry

Cells were obtained from each group, and the culture medium was discarded. Then, trypsin was added for digestion, and 195 μL of Annexin V-FITC (Sigma-Aldrich, USA) binding solution was added for cell re-suspension. After staining with 5 μL of Annexin V-FITC and 10 μL of PI, a flow cytometer was used to measure the cell apoptosis rate.

### Statistical analysis

The data were analyzed statistically using SPSS 24.0 software. Normally distributed data were expressed as (ࣥχ±s). Analysis of variance and LSD post hoc tests were used for comparison among multiple groups. Non-normally distributed data were represented by the median (interquartile range), with the Kruskal-Wallis H test for comparison; p *<* 0.05 was considered statistically significant.

## Results

### Comparison of behavior test results

**Fig. 1:**
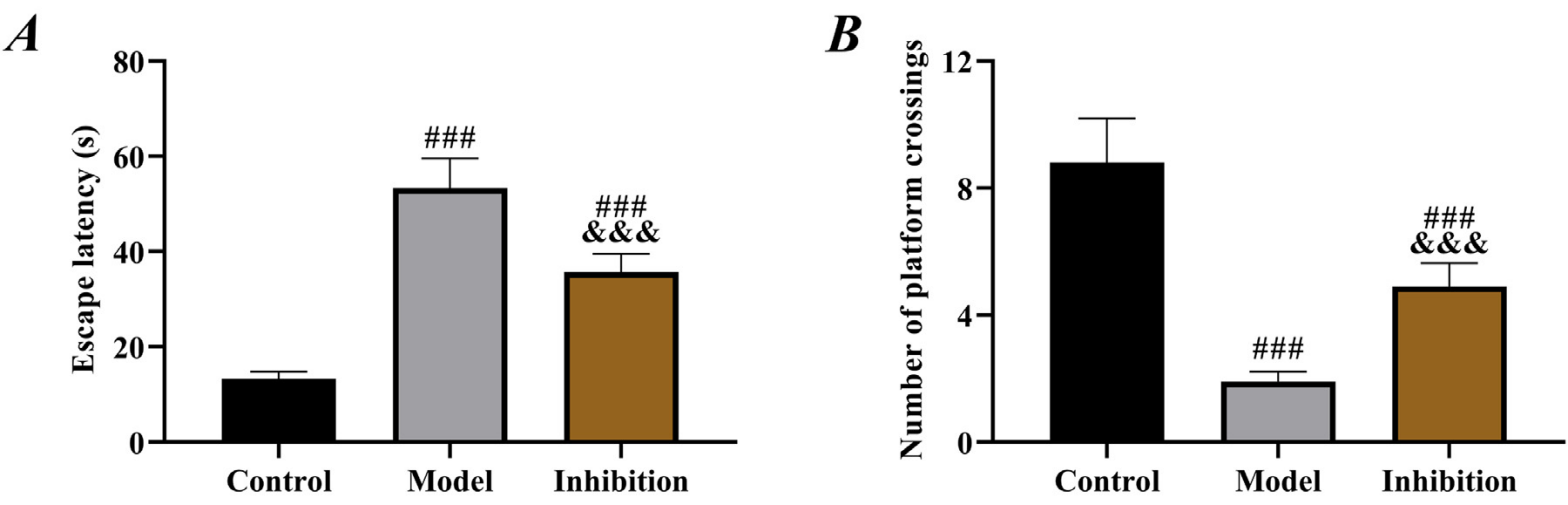
**Behavior test results.** (A) comparison of escape latency, (B) comparison of number of platform crossings. Note: ^###^ p < 0.05 compared with control group, ^&&&^ p < 0.05 compared with model group.

Compared to rats in the control group, those in both the model and inhibition groups showed significantly prolonged escape latency and decreased number of platform crossings (p < 0.05). Furthermore, rats in the inhibition group showed shorter escape latency and an increased number of platform crossings than those in the model group (p < 0.05, Fig. 1).

### Comparison of OS results

**Fig. 2:**
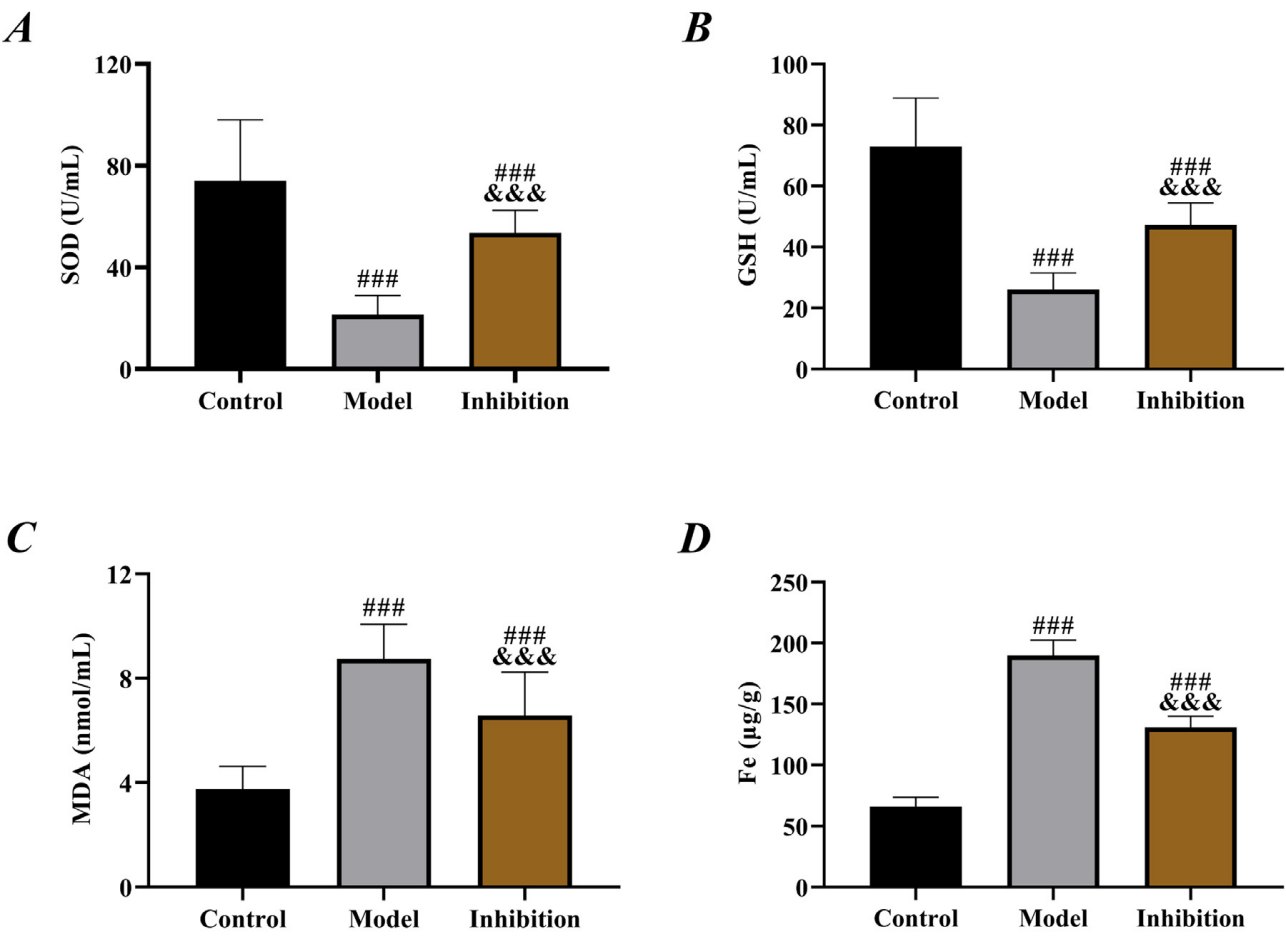
**OS results.** (A) comparison of SOD, (B) comparison of GSH, (C) comparison of MDA, (B) comparison of Fe content. Note: ^###^ p < 0.05 compared with control group, ^&&&^ p < 0.05 compared with model group.

Compared to the control group, both the model and inhibition groups exhibited reduced SOD and GSH while elevated MDA (p < 0.05). Specifically, SOD and GSH were higher while MDA was lower in the inhibition group than in the model group (p < 0.05). Additionally, the Fe content in the three groups of rats ranked from highest to lowest as follows: the model group, the inhibition group, the control group (p < 0.05, Fig. 2).

### Comparison of Keap1/Nrf2 pathway status

**Fig. 3:**
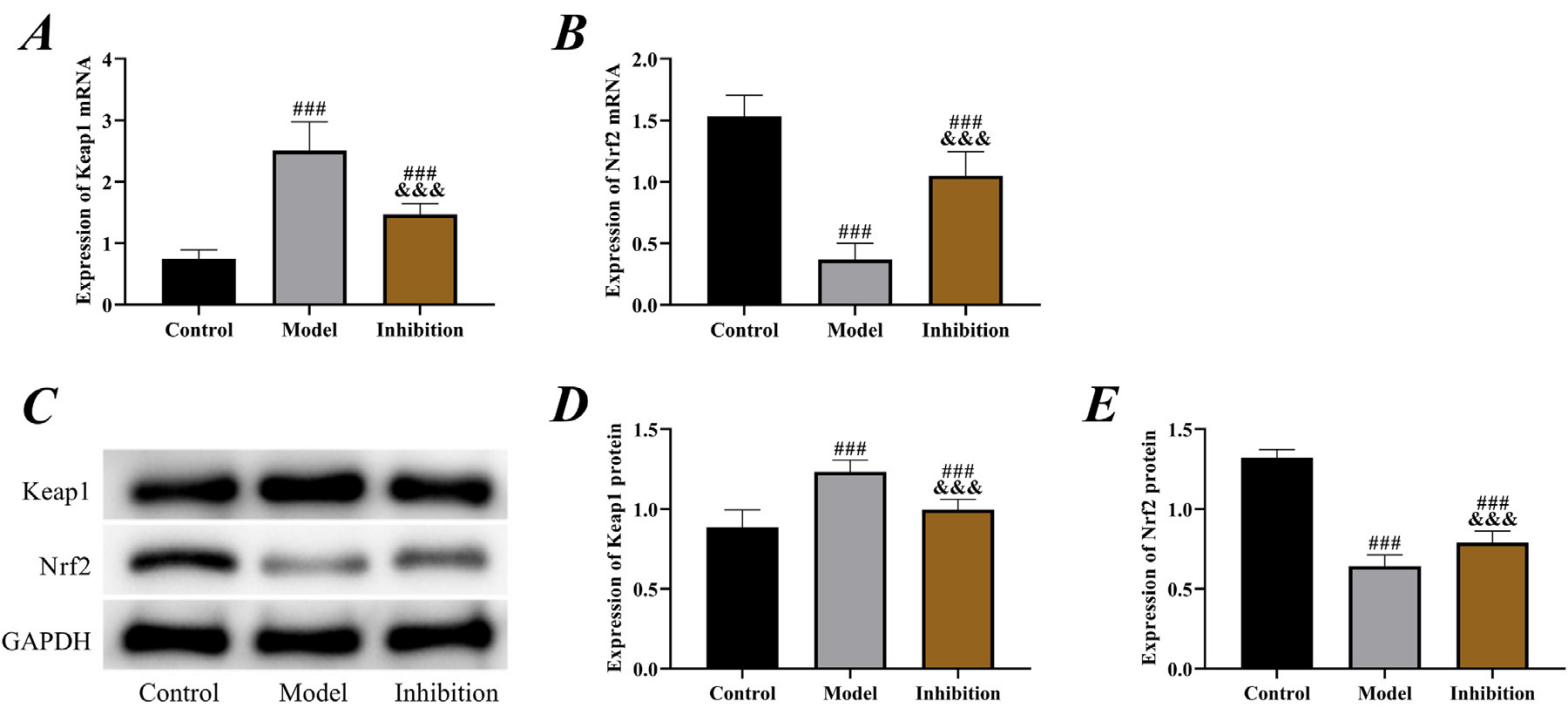
**Keap1/Nrf2 pathway status.** (A) PCR was performed to detect the expression of Keap1 mRNA, (B) PCR was performed to detect the expression of Nrf2 mRNA, (C) Western blot, (D) expression of Keap1 protein. (E) expression of Nrf2 protein. Note: ^###^ p < 0.05 compared with control group, ^&&&^ p < 0.05 compared with model group.

According to the results of PCR and Western blot, the expression of Keap1 was significantly higher while the expression of Nrf2 was lower in the model group compared to the control group (p < 0.05), indicating that the Keap1/Nrf2 pathway was activated in EP. Furthermore, in the inhibition group, the expression of Keap1 was notably reduced while Nrf2 was increased compared to the model group (p < 0.05), indicating that ATRA had a significant inhibitory effect, which successfully blocked the expression of Keap1 and activated Nrf2 (Fig. 3).

### Comparison of cell ferroptosis

**Fig. 4:**
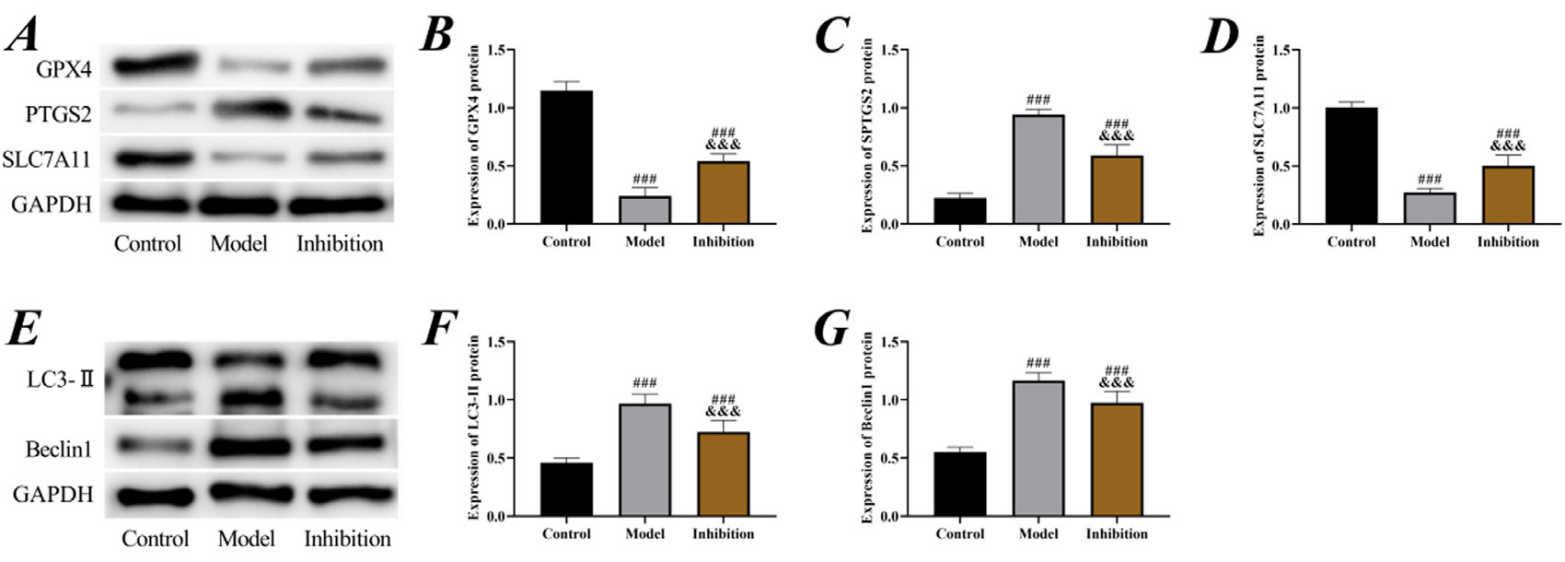
**Cell ferroptosis.** (A) Western blot of ferroptosis proteins, (B) expression of GPX4 protein, (C) expression of PTGS2 protein, (D) expression of SLC7A11 protein, (E) Western blot of autophagy proteins, (F) expression of LC3-II protein, (G) expression of Beclin1 protein. Note: ^###^ p < 0.05 compared with control group, ^&&&^ p < 0.05 compared with model group.

Subsequently, the expression of ferroptosis-associated proteins was examined. It was observed that the expression of GPX4 and SLC7A11 proteins in both the model group and the inhibition group was lower than that in the control group, while the expression of PTGS2, LC3-II, and Beclin1 was higher (p < 0.05), suggesting the presence of significant ferroptosis in EP rats. Compared to the model group, the expression of GPX4 and SLC7A11 increased while PTGS2, LC3-II, and Beclin1 decreased in the inhibition group (p < 0.05), suggesting that the ferroptosis was blocked (Fig. 4).

### Comparison of cell viability

**Fig. 5:**
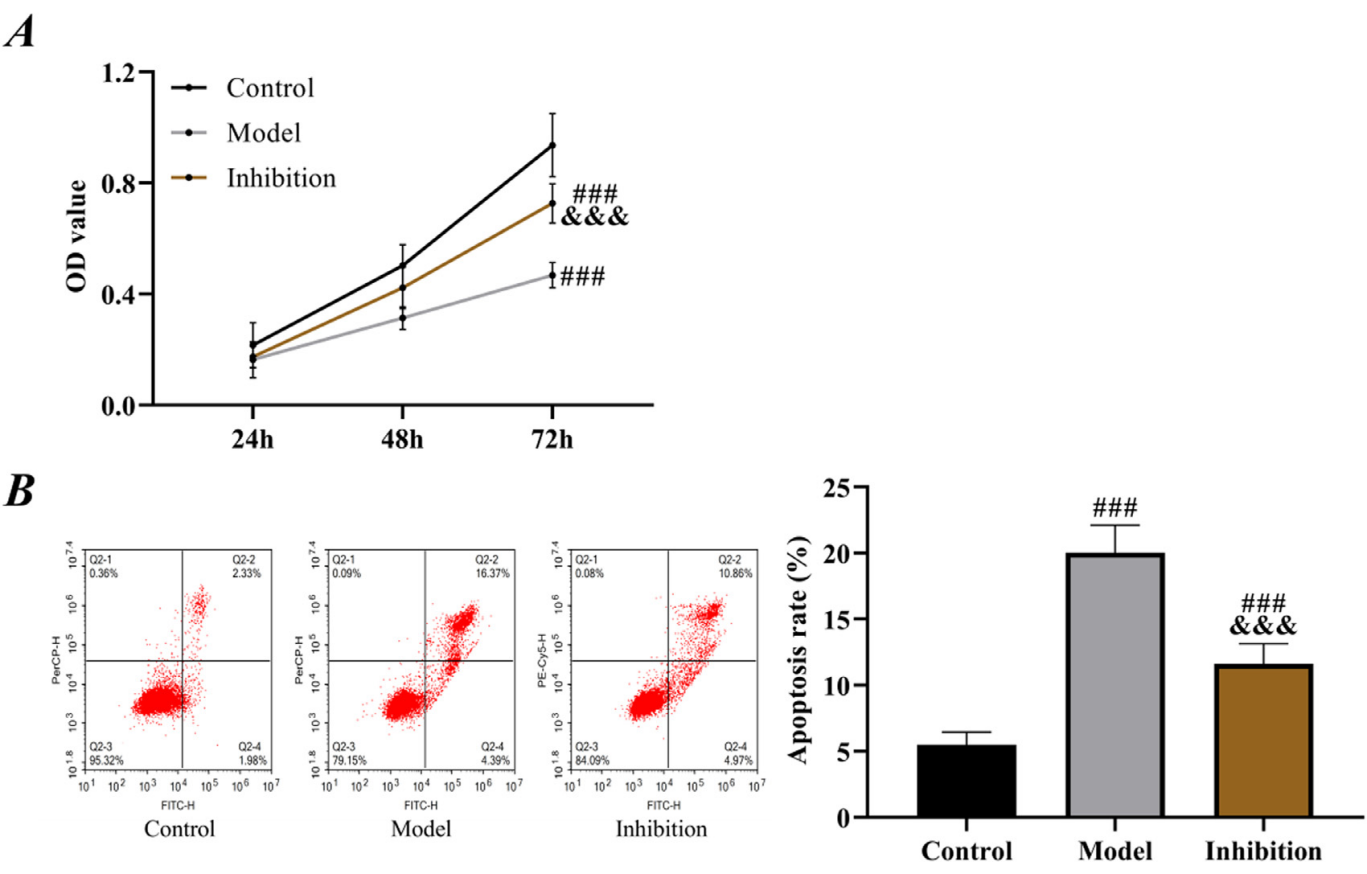
**Cell viability.** (A) cell growth curves, (B) cell apoptosis rate. Note: ^###^ p < 0.05 compared with control group, ^&&&^ p < 0.05 compared with model group.

Compared with the control group, the model group showed remarkably lower proliferation ability and higher apoptosis rate than the control group (p < 0.05). For the inhibition group, its proliferation ability was lower than that of the control group and higher than that of the model group; its apoptosis rate was higher than that of the control group and lower than that of the model group (p < 0.05, Fig. 5).

## Discussion

Currently, the incidence of EP is showing an increasing trend year by year. Although EP progression in 80% of patients can be controlled through treatment, a small percentage of patients still have a poor prognosis and experience recurrent episodes.^18^ Since EP cannot be cured at present, patients will face the potential risk of recurrence at any time once they stop taking the medication.^19^ Molecular immunotherapy is the key to the diagnosis and treatment of various diseases in recent years, a thorough understanding and mastery of the molecular mechanism is therefore essential.^20^ In this study, the authors found that the Keap1/Nrf2 pathway was closely related to EP and regulated OS and ferroptosis in EP, suggesting that the Keap1/Nrf2 pathway may be important in future clinical treatment of EP.

To identify the relationship between the Keap1/Nrf2 pathway and EP, the authors established an EP rat model by pentylenetetrazol, which is currently the most commonly used method for establishing an *in vitro* EP model in clinical research, and its effectiveness has been validated multiple times.^21^^,^^22^ By examining the status of the Keap1/Nrf2 pathway in rats from different groups, it was observed that the Keap1 was elevated while the Nrf2 was reduced in the model group, indicating that the Keap1/Nrf2 pathway was activated in EP. Keap1 is one of the main factors regulating the level of Nrf2 in cells; it interacts with Nrf2 and maintains its stability.^23^ The cysteine residues of Keap1 lose ubiquitin activity due to the continuous production of Reactive Oxygen Species (ROS) under stimulation by oxidative conditions, leading to the translocation of Nrf2 from the cytoplasm to the nucleus, where it binds to ARE and initiates the expression of downstream antioxidant genes such as HO-1.^24^ Therefore, the Keap1/Nrf2 pathway is also considered one of the most important pathways in OS, which is involved in the treatment of cardiovascular disorders, nervous system disorders, tumors, and other diseases.^25^ Decreased Keap1 while increased Nrf2 in the inhibition group indicated a successful intervention in the expression of the pathway.

Prolonged escape latency and reduced number of platform crossings in the model group in behavior tests indicated obvious neurological disorders in rats, in line with the pathological manifestation of EP,^26^ which preliminarily confirmed successful modeling. Compared with the model group, rat behavior was significantly improved in the inhibition group, with shortened escape latency and increased number of platform crossings, indicating effective restoration of neural function, which preliminarily suggests that inhibition of the Keap1/Nrf2 pathway reverses the neurological disorders in rats with EP. In the model group, SOD and GSH were reduced but MDA was elevated, confirming the presence of significant OS. Enhanced OS in patients with EP is a well-established view in the clinic and has been repeatedly mentioned in several previous studies. In contrast, the inhibition group showed notably increased SOD and GSH and decreased MDA compared to the model group, indicating enhanced antioxidant capacity and reduced oxidative damage of rats in this group. Multiple previous studies have mentioned that inhibiting Keap1 to activate the Nrf2 pathway alleviates OS in rats with diabetic cataracts and osteoarthritis.^27^^,^^28^ Therefore, the results above are expected. EP leads to depolarization of mitochondrial membranes, increased production of free radicals and ROS, decreased activity of antioxidant enzymes such as SOD, GSH and elevated levels of MDA, Fe (a final product of membrane lipid metabolism), thereby resulting in cell damage.^29^ Therefore, the above results also suggest that immunotherapy targeting the Keap1/Nrf2 pathway may be a new research direction for EP treatment in the future.

Neuron ferroptosis is another focus of attention in the pathological damage process of EP. Studies have shown that ferroptosis inducers directly or indirectly affect GPX4, leading to the accumulation of intracellular ROS and triggering cell death mechanisms through OS.^30^ Here in the model group, decreased GPX4 and SLC7A11, increased PTGS2, LC3-II, and Beclin1, lowered neuronal viability and elevated apoptosis were observed, confirming that EP promoted excessive ferroptosis of neurons. Significant improvement in ferroptosis in the inhibition again verified that inhibition of the Keap1/Nrf2 pathway reversed neuron ferroptosis in EP. GPX4, SLC7A11, and PTGS2 are the most typical ferroptosis marker proteins, and when GPX4 and SLC7A11 are decreased and PTGS2 is elevated, it indicates that excessive ferroptosis is occurring and the normal life cycle of the cell is shortened.^31^ LC3-II and Beclin1, as autophagy marker proteins, are likewise a pathological manifestation of excessive cellular ferroptosis.^32^ For EP, the increased ferroptosis and autophagy in neuronal cells is exactly the pathological manifestation of their memory, cognitive, and neurological function impairments.^33^ GPX4 and Nrf2 play a negative regulatory role in ferroptosis by respectively limiting ROS production and reducing cellular iron uptake.^34^ Therefore, after activation of Nrf2 by the expression of Keap1 in tissues, the cellular iron uptake capacity is enhanced and the cellular stability is more reliably guaranteed, so that the process of ferroptosis is significantly blocked. Such a point is also confirmed by the significant reduction in Fe content in the hippocampus of the inhibition group compared to the model group. Similarly, Zhao S et al. also indicated in their study that inhibition of the Keap1/Nrf2 pathway blocked ferroptosis after acute liver damage,^35^ which again supports these results.

However, due to the inherent differences between animal models and the human body, clinical trials are urgently needed to demonstrate the relationship between the Keap1/Nrf2 pathway and EP. Additionally, more experiments (e.g., cell cycle analysis, and pathological changes in rat brain tissues) are needed to confirm the mechanisms and pathways through which the Keap1/Nrf2 pathway affects EP. In the future, the authors will also conduct supplementary experiments to address these shortcomings, so as to improve the analysis of the impact of the Keap1/Nrf2 pathway on EP.

## Conclusion

The progress of the Keap1/Nrf2 pathway is closely related to EP. Inhibition of the Keap1/Nrf2 pathway reverses OS and neuron ferroptosis in EP rats. In the future, immunotherapy targeting the inhibition of the Keap1/Nrf2 pathway may become a promising new treatment option for EP, which ensures the prognosis and health of patients with EP.

## Conflicts of interest

The authors declare no conflicts of interest.

## References

[1] Falco-Walter J (2020). Epilepsy-Definition, Classification, Pathophysiology, and Epidemiology. Semin Neurol.

[2] Kanner AM, Bicchi MM. (2022). Antiseizure Medications for Adults With Epilepsy: A Review. JAMA.

[3] Perucca P, Bahlo M, Berkovic SF. (2020). The Genetics of Epilepsy. Annu Rev Genomics Hum Genet.

[4] Rugg-Gunn F, Miserocchi A, McEvoy A. (2020). Epilepsy surgery. Pract Neurol.

[5] Specchio N, Wirrell EC, Scheffer IE, Nabbout R, Riney K, Samia P (2022). International League Against Epilepsy classification and definition of epilepsy syndromes with onset in childhood: Position paper by the ILAE Task Force on Nosology and Definitions. Epilepsia.

[6] Bellezza I, Giambanco I, Minelli A, Donato R. (2018). Nrf2-Keap1 signaling in oxidative and reductive stress. Biochim Biophys Acta Mol Cell Res.

[7] Guo Z, Mo Z. (2020). Keap1-Nrf2 signaling pathway in angiogenesis and vascular diseases. J Tissue Eng Regen Med.

[8] Yamamoto M, Kensler TW, Motohashi H. (2018). The KEAP1-NRF2 System: a Thiol-Based Sensor-Effector Apparatus for Maintaining Redox Homeostasis. Physiol Rev.

[9] Ding X, Jian T, Wu Y, Zuo Y, Li J, Lv H (2019). Ellagic acid ameliorates oxidative stress and insulin resistance in high glucose-treated HepG2 cells via miR-223/keap1-Nrf2 pathway. Biomed Pharmacother.

[10] Chen S, Chen Y, Zhang Y, Kuang X, Liu Y, Guo M (2020). Iron Metabolism and Ferroptosis in Epilepsy. Front Neurosci.

[11] Bie B, Wang Z, Chen Y, Sheng L, Li You HH (2021). Vagus nerve stimulation affects inflammatory response and anti-apoptosis reactions via regulating miR-210 in epilepsy rat model. Neuroreport.

[12] Kishore M, Pradeep M, Narne P, Jayalakshmi S, Panigrahi M, Patil A (2023). Regulation of Keap1-Nrf2 axis in temporal lobe epilepsy-hippocampal sclerosis patients may limit the seizure outcomes. Neurol Sci.

[13] Hu QP, Yan HX, Peng F, Feng W, Chen F-F, Huang X-Y (2021). Genistein protects epilepsy-induced brain injury through regulating the JAK2/STAT3 and Keap1/Nrf2 signaling pathways in the developing rats. Eur J Pharmacol.

[14] Van Erum J, Van Dam D, De Deyn PP. (2019). PTZ-induced seizures in mice require a revised Racine scale. Epilepsy Behav.

[15] Othman MZ, Hassan Z, Che Has AT (2022). Morris water maze: a versatile and pertinent tool for assessing spatial learning and memory. Exp Anim.

[16] Lee HY, Ryu GH, Choi WY, Yang WS, Lee HW, Ma CJ. (2018). Protective Effect of Water Extracted Spirulina maxima on Glutamate-induced Neuronal Cell Death in Mouse Hippocampal HT22 Cell. Pharmacogn Mag.

[17] Wang XJ, Hayes JD, Henderson CJ, Wolf CR. (2007). Identification of retinoic acid as an inhibitor of transcription factor Nrf2 through activation of retinoic acid receptor alpha. Proc Natl Acad Sci U S A.

[18] Loscher W, Potschka H, Sisodiya SM, Vezzani A. (2020). Drug Resistance in Epilepsy: Clinical Impact, Potential Mechanisms, and New Innovative Treatment Options. Pharmacol Rev.

[19] Piper RJ, Richardson RM, Worrell G, Carmichael DW, Baldeweg T, Litt B (2022). Towards network-guided neuromodulation for epilepsy. Brain.

[20] Huang Q, Liu J, Shi Z, Zhu X. (2020). Correlation of MMP-9 and HMGB1 expression with the cognitive function in patients with epilepsy and factors affecting the prognosis. Cell Mol Biol (Noisy-le-grand).

[21] Cetindag Ciltas A, Ozdemir E, Gumus E, Taskiran AS, Gunes H, Arslan G (2022). The Anticonvulsant Effects of Alpha-2 Adrenoceptor Agonist Dexmedetomidine on Pentylenetetrazole-Induced Seizures in Rats. Neurochem Res.

[22] Sahin H, Erbas O. (2023). Beneficial effects of sennoside B on pentylenetetrazole-induced seizures in rats. Hum Exp Toxicol.

[23] Cuadrado A, Rojo AI, Wells G, Hayes JD, Cousin SP, Rumsey WL (2019). Therapeutic targeting of the NRF2 and KEAP1 partnership in chronic diseases. Nat Rev Drug Discov.

[24] Liu Y, Tao S, Liao L, Li Y, Li H, Li Z (2020). TRIM25 promotes the cell survival and growth of hepatocellular carcinoma through targeting Keap1-Nrf2 pathway. Nat Commun.

[25] Fu D, Wang C, Yu L, Yu R. (2021). Induction of ferroptosis by ATF3 elevation alleviates cisplatin resistance in gastric cancer by restraining Nrf2/Keap1/xCT signaling. Cell Mol Biol Lett.

[26] Valassina N, Brusco S, Salamone A, Serra L, Luoni M, Giannelli S (2022). Scn1a gene reactivation after symptom onset rescues pathological phenotypes in a mouse model of Dravet syndrome. Nat Commun.

[27] Dempke WCM, Reck M. (2021). KEAP1/NRF2 (NFE2L2) mutations in NSCLC - Fuel for a superresistant phenotype?. Lung Cancer.

[28] Bollong MJ, Lee G, Coukos JS, Yun H, Zambaldo C, Chang JW (2018). A metabolite-derived protein modification integrates glycolysis with KEAP1-NRF2 signalling. Nature.

[29] Adelusi TI, Du L, Hao M, Zhou X, Xuan Q, Apu C (2020). Keap1/Nrf2/ARE signaling unfolds therapeutic targets for redox imbalanced-mediated diseases and diabetic nephropathy. Biomed Pharmacother.

[30] Yang W, Wang Y, Zhang C, Huang Y, Yu J, Shi L (2022). Maresin1 Protect Against Ferroptosis-Induced Liver Injury Through ROS Inhibition and Nrf2/HO-1/GPX4 Activation. Front Pharmacol.

[31] Cai Y, Yang Z. (2021). Ferroptosis and Its Role in Epilepsy. Front Cell Neurosci.

[32] Gao W, Wang X, Zhou Y, Wang X, Yu Y. (2022). Autophagy, ferroptosis, pyroptosis, and necroptosis in tumor immunotherapy. Signal Transduct Target Ther.

[33] Yang N, Guan Q-W, Chen F-H, Xia Q-X, Yin X-X, Zhou H-H (2020). Antioxidants Targeting Mitochondrial Oxidative Stress: Promising Neuroprotectants for Epilepsy. Oxid Med Cell Longev.

[34] Shin D, Kim EH, Lee J, Roh JL. (2018). Nrf2 inhibition reverses resistance to GPX4 inhibitor-induced ferroptosis in head and neck cancer. Free Radic Biol Med.

[35] Zhao S, Huang M, Yan L, Zhang H, Shi C, Liu J (2022). Exosomes Derived from Baicalin-Pretreated Mesenchymal Stem Cells Alleviate Hepatocyte Ferroptosis after Acute Liver Injury via the Keap1-NRF2 Pathway. Oxid Med Cell Longev.

